# Outcomes of Retzius-sparing versus conventional robot-assisted radical prostatectomy: A KSER update series systematic review and meta-analysis

**DOI:** 10.1371/journal.pone.0268182

**Published:** 2022-05-26

**Authors:** Doo Yong Chung, Hae Do Jung, Do Kyung Kim, Min Ho Lee, Sin Woo Lee, Sunghyun Paick, Joo Yong Lee, Seung Hyun Jeon

**Affiliations:** 1 Department of Urology, Inha University School of Medicine, Incheon, Korea; 2 Department of Urology, Wonkwang University Sanbon Hospital, Wonkwang University School of Medicine, Gunpo, Korea; 3 Department of Urology, Soonchunhyang University Hospital, Soonchunhyang University College of Medicine, Seoul, Korea; 4 Department of Urology, Gyeongsang National University Changwon Hospital, Gyeongsang National University School of Medicine, Changwon, Korea; 5 Department of Urology, Eulji General Hospital, Eulji University School of Medicine, Seoul, Korea; 6 Department of Urology, Konkuk University School of Medicine, Seoul, Korea; 7 Department of Urology, Severance Hospital, Urological Science Institute, Yonsei University College of Medicine, Seoul, Korea; 8 Center of Evidence Based Medicine, Institute of Convergence Science, Yonsei University, Seoul, Korea; 9 Department of Urology, Kyung Hee University School of Medicine, Seoul, Korea; Imperial College Healthcare NHS Trust, UNITED KINGDOM

## Abstract

**Background:**

Robotic‐assisted radical prostatectomy(RARP) is widely used to surgically treat of localized prostate cancer. Among RARP, retzius-sparing techniques(RS-RARP) are implemented through douglas pouch, not the existing conventional approach(C-RARP). We conducted an updated systematic review and meta-analysis including recent published papers.

**Materials & methods:**

Systematic review was performed following the PRISMA guideline. PubMed, EMBASE, and Cochrane Library were searched up to August 2021. We conducted meta-analysis as follows; Participants, patients with biopsy-proven PCa; Interventions, Patients underwent C-RARP or RS-RALP; Outcomes, comparison of continence recovery rate, positive surgical margins(PSM), complication, operation time and estimated blood loss(EBL) included for analysis.

**Results:**

Thirteen studies with a total of 2917 patients were included for meta-analysis. Among them, three were randomized controlled trials (RCT) studies and the rest were non-RCT studies. Incontinence was analyzed with zero pad and safety pad, respectively. There showed a statistically significant advantage for RS-RARP in terms of continence recovery at 1 month(0 pad; OR 0.28, (0.16–0.47), safety-pad; OR 0.12 (0.07–0.22), p<0.001), as well as at 3 months(0 pad; OR 0.31 (0.18–0.53), safety-pad; OR 0.23 (0.14–0.40) p<0.001), 6 months(0 pad; OR 0.29 (0.17–0.51), safety-pad; OR 0.13 (0.06–0.27), p<0.001). And after 12 months, RS-RARP showed better results only in the safety-pad(0 pad; OR 0.64 (0.35–1.18), p = 0.15, safety-pad; OR 0.12 (0.04–0.36), p<0.001). In PSM, there was no statistical difference between two group at overall stage, but RS-RARP was observed to be higher than C-RARP in pT3 subgroup analysis(OR 0.74 (0.55–0.99), p = 0.047) (Fig 1). Whereas, there was no significant difference between the two groups in complication, operation time, and EBL.

**Conclusions:**

Our analysis showed that RS-RARP is superior about early continence recovery than C-RARP. However, RS-RARP showed relatively high PSM in locally advanced PCa of pT3 or above. Therefore, although RS-RARP has few advantages about functional outcomes, we think that caution should be exercised when approaching patients with high-risk local diseases.

## 1. Introduction

Prostate cancer (PCa) is the most common type of newly diagnosed malignancy in men, accounting for nearly 20% of all diagnosed cancers among men in the United State in 2020 [1]. Radical prostatectomy (RP) is one of the most commonly used therapeutic approaches for localized and locally advanced PCa. Robot-assisted RP (RARP) was first introduced in 2001 [2]. RARP has been widely used recently because of its advantages such as shorter hospital stay, less surgical trauma, and lesser need for analgesics than the existing open technique [3, 4]. However, despite advancements in surgical experience and technology, functional complications such as urinary incontinence and erectile dysfunction caused by RP are the urologist’s conundrum. Therefore, continuous technological developments aim to reduce its associated functional complications. Accordingly, many urologists have devised and implemented methods to prevent incontinence, such as membranous urethral length preservation, posterior musculofascial reconstruction, and bladder neck preservation [5–8]. In 2010, Bocciardi et al. [9] published a new approach–Retzius-sparing RARP (RS-RARP)–that passes through the pouch of Douglas and spares the Retzius structures involved in continence. In a follow-up presentation, they reported remarkable results with no patients using safety liners by 1 year postoperative. This new surgical method has since been implemented by many surgeons and is being compared to conventional -RARP (C-RARP). Existing meta-analyses on this topic have been published, but many papers were not included, thus results such as continence and positive surgical margin (PSM) are conflicting, and the evidence level tends to be low. Therefore, here we included all recently published studies and compared the updated results of RS-RARP and C-RARP in a systematic review and meta-analysis.

## 2. Materials and methods

### 2.1 Search strategy and data extraction

This study was conducted in accordance with the Preferred Reporting Items for Systematic Reviews and Meta-Analyses (PRISMA) statement (http://www.prisma-statement.org/) [10]. Relevant studies that compared the two robotic surgical methods (C-RARP and RS-RARP) for localized and locally advanced PCa cases were searched up to July 2021 using PubMed, Ovid-EMBASE, and the Cochrane Central Register of Controlled Trials using the following Medical Subject Headings terms: “prostate cancer,” “prostate carcinoma,” “Retzius,” “sparing,” “prostatectomy,” and relevant variations. We limited the search to studies published in English. Two reviewers (DYC and HDJ) independently reviewed the titles and abstracts of the retrieved articles based on the inclusion criteria. Any discrepancies between them were resolved by a third reviewer (JYL). Because this study was a systematic review and meta-analysis, it was exempted from ethics committee or institutional review board approval.

### 2.2 Inclusion criteria and study eligibility

The eligibility of each study was assessed considering participants, interventions, comparators, outcomes, and study design approach [11]: participants, patients with biopsy-proven PCa who had localized or locally advanced PCa without neoadjuvant therapy; interventions, PCa patients who underwent C-RARP; comparators, PCa patients with the same characteristics who underwent RS-RALP; outcomes, incontinence recovery rates, PSM, estimated blood loss (EBL), operation time, complication rates; and study design, no restrictions on research design, with both randomized controlled trials (RCTs) and non-RCTs included in the analysis.

The primary endpoint was incontinence recovery rates, the secondary endpoint was PSM, and the tertiary endpoints were EBL, operation time, and complication rates. The definition of urinary incontinence recovery was divided into zero pads and safety pads and analyzed. Safety pad was defined as one security pad per day used in most studies. For PSM, a sub-analysis was performed for each T stage as well as for the overall analysis.

### 2.3 Quality assessment

Quality assessments were performed independently by two reviewers (DYC and DKK) using the Cochrane risk of bias tool and the Newcastle–Ottawa Scale [12, 13]. The Cochrane risk of bias tool for the quality assessment of RCTs was recommended by the Cochrane Handbook for Systematic Reviews of Interventions and includes the following risk of bias domains: (1) random sequence generation, (2) allocation concealment, (3) blinding of participants and personnel, (4) blinding of outcomes assessment, (5) incomplete outcomes data, (6) selective reporting, and (7) other potential biases. Each item was further divided into three categories based on the risk of bias: high, low, and unknown.

For the Newcastle–Ottawa Scale, a tool for evaluating non-RCTs, the three major assessment categories were selection, comparability, and exposure. Studies can be rated as up to nine stars [14]. A final score of six stars or more indicates high quality.

### 2.4 Statistical analysis

Odds ratios (ORs) and 95% confidence intervals (CI) were calculated for dichotomous variables. The weighted mean difference (WMD) was calculated for continuous variables. Inter-study heterogeneity was assessed using chi-square and I^2^ tests. A Cochran Q statistic p value <0.05 or an I^2^ statistic >50% was used to indicate statistically significant heterogeneity between trials [15]. Based on the degree of heterogeneity, a random-effects or fixed-effects model was applied to calculate summary measures [16]. The meta-analysis was conducted using Review Manager Version 5.3 (RevMan, The Nordic Cochrane Center, The Cochrane Collaboration, 2013) and R (version 4.1.1, R Foundation for Statistical Computing, Vienna, Austria; http://www.r-project.org/) and its meta and metafor packages. A nonsignificant test result (p>0.05) indicated that the test hypothesis is true or should be accepted [17]. In the analysis of less than 10 studies, funnel plots were not used; only analyses containing 10 or more studies were added (S1 Fig) [18].

## 3. Results

### 3.1 Systematic review process

**Fig 1:**
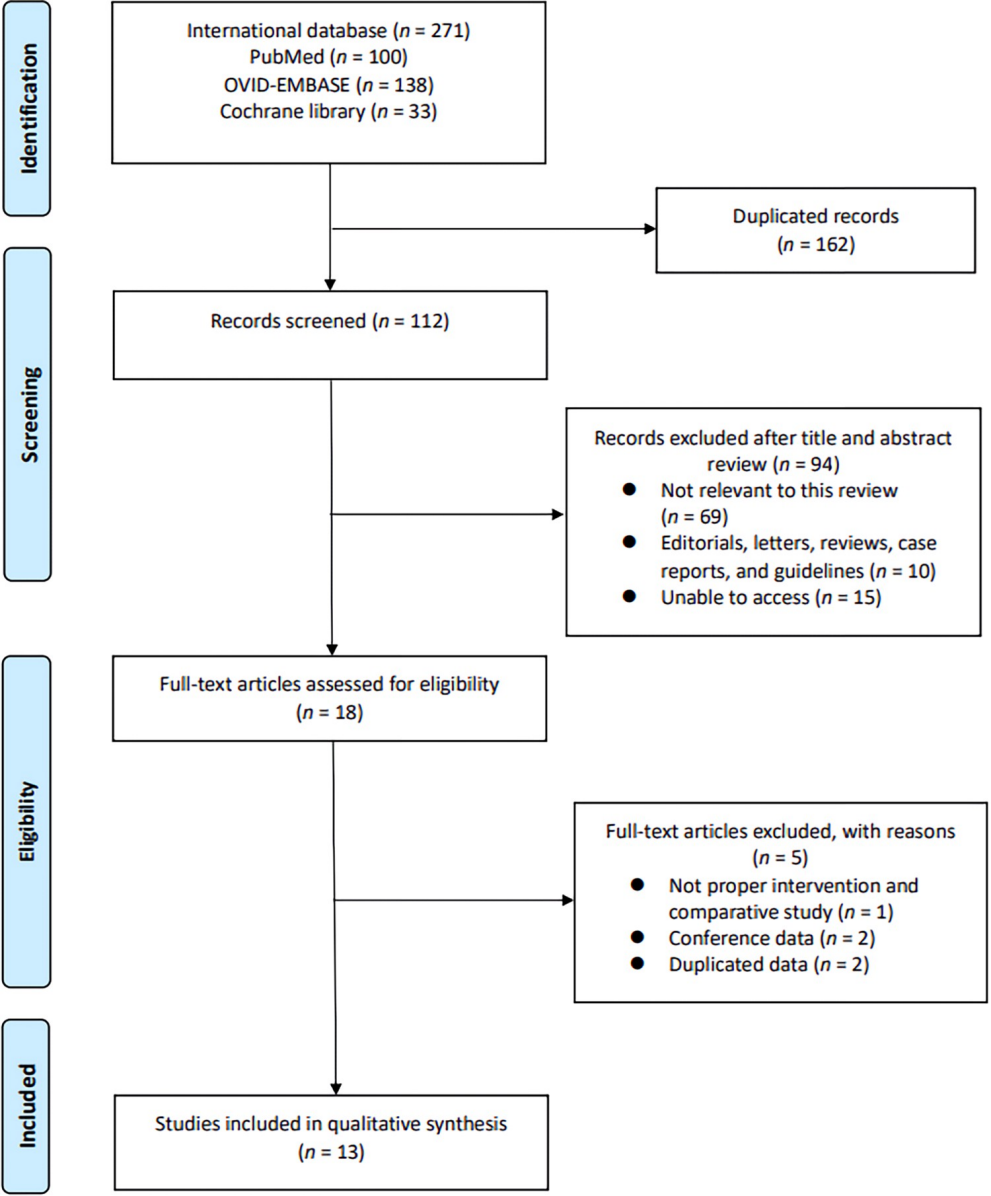
Study selection flowchart according to Preferred Reporting Items for Systematic Reviews and Meta-analysis guidelines.

The PRISMA guidelines were followed; a flowchart of the study selection process is shown in Fig 1. The initial international database search identified 271 studies (100 in PubMed, 138 in OVID-EMBASE, and 33 in the Cochran Library). Of them, 162 remained after the removal of duplicates. After the title and abstract screening, 94 articles were excluded. Subsequently, 18 full-text articles were evaluated based on pre-established inclusion criteria. As a result, a total of 13 papers [19–32] (2,917 patients) were included in the final analysis (Table 1). Two studies included the same patients. Therefore, the two were combined included in the meta-analysis [19, 20].

**Table 1:** Characteristics of the eligible studies.

Authors (Year)	Country	Study design	Operation type	Total patients	Continence definition	Incontinence recovery (N, (%))						PSM (event N/ Total N)			Operation or console time	EBL	Complication (overall)
						Imme-diate	1m	3m	6m	9m	12m	T2	T3	Overall	mean±SD Median (IRQ)	mean±SD Median (IRQ)	Event N /Total N
Dalela, Menon et al., (2017) [19, 20]	USA	Randomized control trial	C-RARP	60	0 pad	9(15)	20(33)	36(60)	44(73)	NR	52(87)	NR	NR	8/60	NR	NR	6/60
					Safety pad	29(48)	40(67)	51(85)	56(93)	NR	56(93)						
			RS-RARP	59	0 pad	25(42)	37(68)	45(76)	55(93)	NR	57(97)	NR	NR	15/60	NR	NR	10/60
					Safety pad	42(71)	50(85)	55(93)	58(98)	NR	59(100)						
Eden et al., (2017) [21]	UK	Retrospective	C-RARP	40	Safety pad	NR	28(70)	NR	NR	NR	NR	2/26	2/14	4/40	223	200	4/40
															(155–266)	(100–330)	
			RS-RARP	40		NR	39(98)	NR	NR	NR	NR	3/18	7/22	10/40	200	200	1/40
															(100–238)	(100–500)	
Ghanem et al. (2017) [22]	Israel	Retrospective	C-RARP	51	0 pad	NR	4(8)	NR	34(67)	NR	47(92)	NR	NR	NR	NR	328±59	15/51
			RS-RARP	51		NR	9(18)	NR	44(86)	NR	47(92)	NR	NR	NR	NR	379±30.2	9/51
Sayyid et al. (2017) [23]	Canada	Retrospective	C-RARP	100	Safety pad	NR	NR	25/88	37/75	39/63	39/57	10/77	11/23	21/100	144	100	6/100
								(28)	(49)	(62)	(68)				(118–171)	(50–150)	
			RS-RARP	100		NR	NR	40/67	39/49	27/31	24/25	11/66	16/34	27/100	120	100	12/100
								(60)	(80)	(87)	(96)				(105–142)	(50–200)	
Chang et al., (2018) [24]	Taiwan	Retrospective propensity score matching	C-RARP	30	Safety pad	NR	NR	NR	NR	NR	28(93)	NR	NR	8/30	210±48.80	268.33±274.96	NR
			RS-RARP	30		NR	NR	NR	NR	NR	30(100)	NR	NR	7/30	213.92±68.82	149.52±108.67	NR
Asimakopoulos et al., (2019) [25]	Italy	Randomized control trial	C-RARP	57	0 pad	NR	27(47)	34(60)	36(63)	NR	NR	2/31	2/9	4/40	163.80±32.80	NR	3/40
			RS-RARP	45		NR	36(80)	40(89)	40(89)	NR	NR	4/22	7/17	11/39	179.80±40.90	NR	1/39
Qiu et al., (2020) [26]	USA	Randomized control trial	C-RARP	55	0 pad	NR	NR	35(64)	49(89)	50(91)	51(93)	1/28	7/27	8/55	135	200	6/55
															(110–155)	(150–400)	
			RS-RARP	55		NR	NR	48(87)	51(93)	51(93)	52(95)	5/33	8/22	13/55	105	200	3/55
															(85–125)	(200–300)	
Liao et al., (2020) [27]	Taiwan	Retrospective	C-RARP	92	Safety pad	24(26)	32(35)	61(66)	77(84)	NR	86(93)	NR	NR	24/92	216.4±56.4	268±299.4	NR
			RS-RARP	41		31(76)	36(88)	39(95)	41(100)	NR	41(100)	NR	NR	13/41	216.90±64.50	156.3±115.2	NR
Lee et al., (2020) [28]	Korea	Retrospective propensity score matching	C-RARP	609	Safety pad	NR	54(9)	NR	468(77)	NR	NR	53/347	84/262	137/609	194±44.00	297.43±220.43	9/609
			RS-RARP	609		NR	274(45)	NR	596(98)	NR	NR	42/370	85/239	127/609	149±41.00	279.59±236.58	7/609
Umari et al., (2021) [29]	Italy	Retrospective propensity score matching	C-RARP	201	Safety pad	68	NR	NR	NR	NR	NR	14/132	14/69	28/201	134±37.86	230.15±129.37	4/21
			RS-RARP	282		198	NR	NR	NR	NR	NR	16/199	28/83	44/282	149±41.00	206.81±124.74	15/282
Egan et al., (2021) [30]	USA	Retrospective	C-RARP	70	0 pad	NR	NR	NR	NR	NR	46/70	NR	NR	24/70	250	128±25.7	6/70
											(66)				(100–388)		
					Safety pad	NR	NR	NR	NR	NR	57/70						
											(81)						
			RS-RARP	70	0 pad	NR	NR	NR	NR	NR	30/41	NR	NR	21/70	100	130±26.1	3/70
											(73)						
					Safety pad	NR	NR	NR	NR	NR	40/41				(75–200)		
											(98)						
Deng et al., (2021) [31]	China	Retrospective propensity score matcing	C-RARP	60	Safety pad	18(30)	NR	35(58)	NR	NR	56(93)	NR	NR	7/60	97.8±50.7	110.0±29.4	9/60
			RS-RARP	60		54(90)	NR	60(100)	NR	NR	60(100)	NR	NR	9/60	110.7±66.4	134.2±27.0	5/60
Ota et al., (2021) [32]	Japan	Retrospective	C-RARP	25	Safety pad	6(24)	7(28)	14(56)	19(76)	23(92)	NR	2/20	3/5	5/25	180	170	7/25
															(155–197)	(92–252)	
			RS-RARP	25		18(72)	22(88)	23(92)	24(96)	25(100)	NR	7/24	0/1	7/25	173	390	8/25
															(156–182)	(252–550)	

C-RARP, conventional robot assisted radical prostatectomy; IQR, Interquartile range; NR, not reported; RS-RARP, retzius-sparing robot assisted radical prostatectomy; SD, standard deviation

• Safety pad was defined as one security pad per day.

• Enrolled patients characteristics in included studies: Comparisons of C-RARP and RS-RARP in localized prostate cancer (cT2-3) without neoadjuvant therapy.

Three studies were RCTs [19, 20, 25, 26]; the others [21, 22, 24, 27–32] were retrospective case-control studies. Among them, four [24, 28, 29, 31] analyzed propensity score matching. All trials enrolled patients diagnosed with PCa who had undergone C-RARP or RS-RARP as the initial treatment.

### 3.2 Quality assessment

The quality assessment results based on the Cochrane risk of bias tool are shown in Table 2A. Due to the limitations of the study topic regarding the comparison of surgical methods, all RCTs [19, 20, 25, 26] had high risk for performance bias and detection bias. In one study [19, 20], the risk of selection bias was high. In this study, C-RARP was selected to treat three patients (one with morbid obesity, one with a very large prostate gland, and one with peritoneal dialysis) (Table 2A). The results of the quality assessment using the Newcastle–Ottawa Scale for non-RCT studies are shown in Table 2B. Nine studies received a score of seven points, indicating high quality. In all non-RCT studies, no major problems were noted, except for the selection of control and non-response rates.

**Table 2:** Results of quality assessment by Cochrane risk of bias tool and Newcastle–Ottawa Scale.

**A. Results of quality assessment of randomized control trial study by the Cochrane risk of bias tool**												
Author(s) (Year)		Random Sequence Generation (Selection Bias)	Allocation Concealment (Selection Bias)			Blinding of Participants and Personnel (Performance Bias)		Blinding of Outcome Assessment (Detection Bias)	Incomplete Outcome Data Addressed (Attrition Bias)		Selective Reporting (Reporting Bias)	Other bias
Dalela, Menon et al., (2017) [19, 20]		Low risk	High risk			High risk		High risk	Low risk		Low risk	Unclear
Asimakopoulos et al., (2019) [25]		Low risk	Low risk			High risk		High risk	Low risk		Low risk	Unclear
Qiu et al., (2020) [26]		Low risk	Low risk			High risk		High risk	Low risk		Low risk	Unclear
**B. Results of quality assessment of nonrandomized studies by the Newcastle–Ottawa Scale**												
Author(s) (Year)	Selection (4)						Comparability (2)		Exposure (3)			**Total score**
	Adequate definition of cases			Representativeness of cases	Selection of controls	Definition of controls	Control for important factor or additional factor		Ascertainment of exposure	Same method of ascertainment for cases and controls	Non-Response rate	
Eden et al., (2017) [21]	1			1	0	1	2		1	1	0	7
Ghanem et al. (2017) [22]	1			1	0	1	2		1	1	0	7
Sayyid et al. (2017) [23]	1			1	0	1	2		1	1	0	7
Chang et al., (2018) [24]	1			1	0	1	2		1	1	0	7
Liao et al., (2020) [27]	1			1	0	1	2		1	1	0	7
Lee et al., (2020) [28]	1			1	0	1	2		1	1	0	7
Umari et al., (2021) [29]	1			1	0	1	2		1	1	0	7
Egan et al., (2021) [30]	1			1	0	1	2		1	1	0	7
Ota et al., (2021) [32]	1			1	0	1	2		1	1	0	7

### 3.3 Functional and perioperative outcomes

#### 3.3.1 Incontinence recovery rate

**Fig 2:**
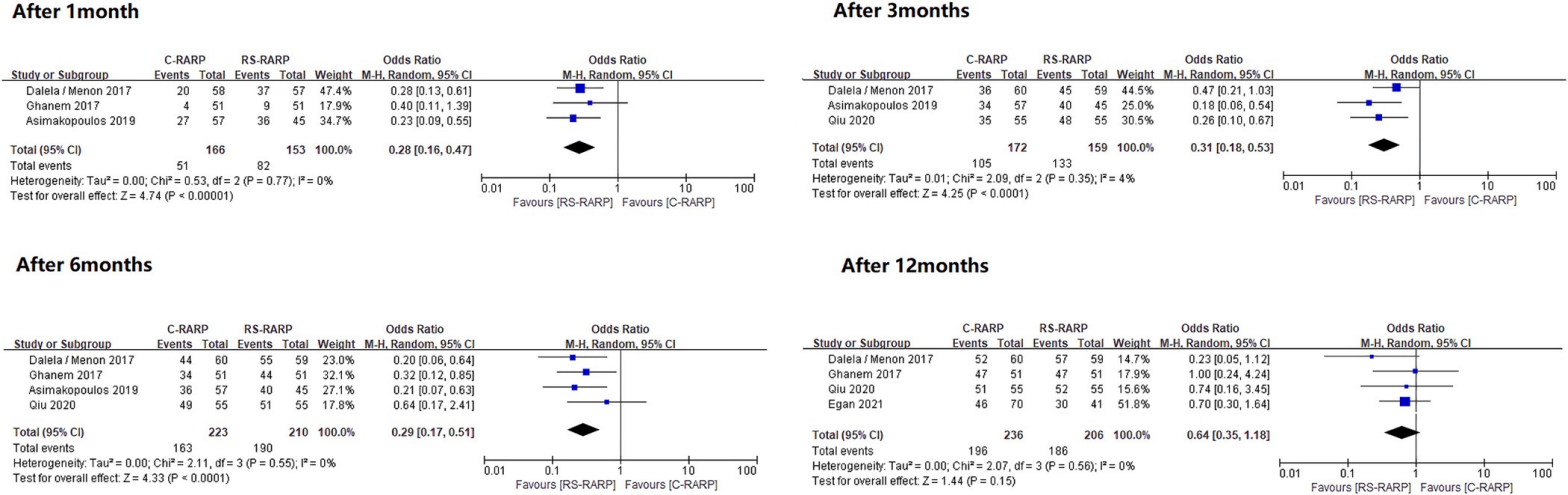
Forest plots of recovery of continence after surgery_zero pad definition.

**Fig 3:**
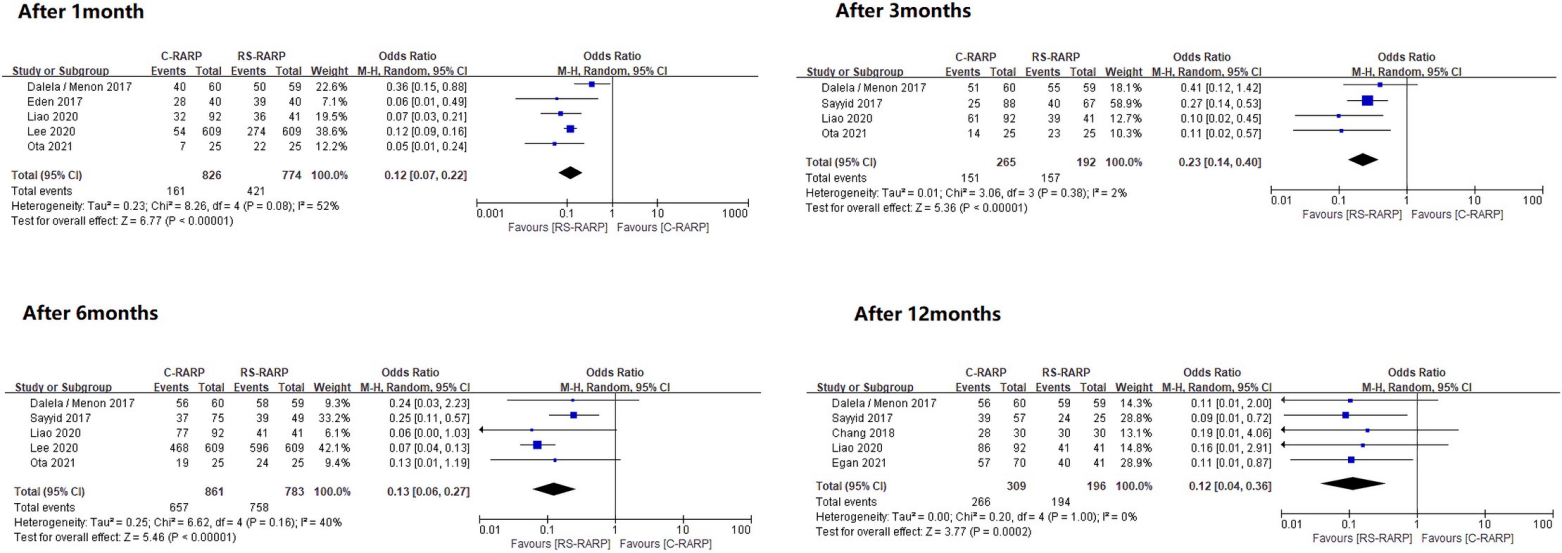
Forest plots of recovery of continence after surgery_safety pad definition.

Urinary incontinence recovery was analyzed in two ways according to the definitions in each study. A forest plot for these analyses were shown in Figs 2 and 3.

- ***Zero pad***

A total of five studies [19, 20, 22, 25, 26, 30] were included in the zero pad analysis. After catheter removal, outcomes at 1, 3, 6, and 12 months were evaluated. Each result was as follows: after 1 month, the outcome was OR, 0.28; 95% CI, 0.16–0.47; p 0.00001; I^2^ = 0%. The 3-month outcome was OR, 0.31; 95% CI, 0.18–0.53; p<0.0001; I^2^ = 4%. The 6-month outcome was OR, 0.29; 95% CI, 0.17–0.51; p<0.0001; I^2^ = 0%. At 1, 3, and 6 months, RS-RALP showed a significantly better incontinence recovery rate than C-RALP. However, at 12 months, the result was OR, 0.64; 95% CI, 0.35–1.18; p = 0.15; I^2^ = 0%. However, this result was not statistically significant. No heterogeneity was observed in any of the analyses (Fig 2).

- ***Safety pad***

A total of 10 studies [19–21, 23, 24, 27–32] were included in the safety pad analysis. Similarly, after catheter removal, outcomes at 1, 3, 6, and 12 months were evaluated. The 1-month outcome was OR, 0.12; 95% CI, 0.07–0.22; p<0.0000; I^2^ = 52%. The 3-month outcome was OR, 0.23; 95% CI, 0.14–0.40; p<0.00001; I^2^ = 2%. The 6-month outcome was OR, 0.13; 95% CI, 0.06–0.27; p<0.00001; I^2^ = 40%. The 12-month outcome was OR, 0.12; 95% CI, 0.04–0.36; p = 0.0002; I^2^ = 0%. At all periods, RS-RALP showed a significantly better incontinence recovery rate than C-RALP. Heterogeneity was observed at the 1-month outcome. No heterogeneity was observed in the remaining outcomes (Fig 3).

#### 3.3.2 Positive surgical margin

A total of 12 studies [19–21, 23–32] involving 2,673 patients were included, and the result of the total patient analysis was OR, 0.88; 95% CI, 0.72–1.06; p = 0.20; I^2^ = 6%. We also performed a sub-analysis of each T stage using studies that reported PSM according to T stage.

**Fig 4:**
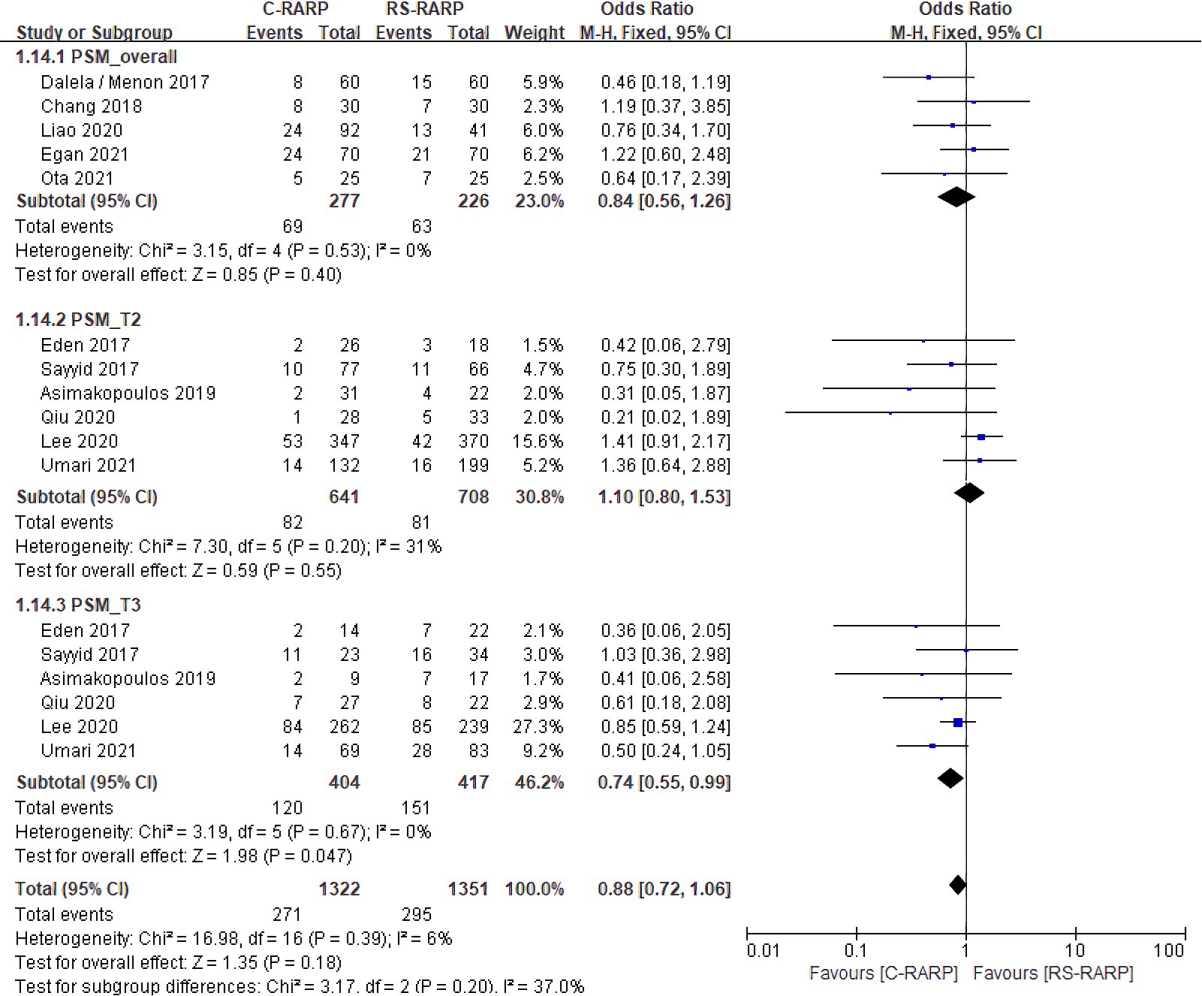
Forest plots of postive surgical margin (overall stage and stratified based on pathological stage).

At the T2 stage, the PSM results were OR, 1.10; 95% CI, 0.80–1.53; p = 0.55; I^2^ = 31%. At the T3 stage, the PSM results were OR, 0.74; 95% CI, 0.55–0.99; p = 0.047; I^2^ = 0%. The overall PSM result of five studies without T stage classification was OR, 0.84; 95% CI, 0.56–1.26; p = 0.40; I^2^ = 0%). In terms of PSM, the study results of the total patient and T2 stage were not statistically significant, but C-RARP showed better results than RS-RARP for the T3 stage (Fig 4).

#### 3.3.3 EBL and operation time

**Fig 5:**
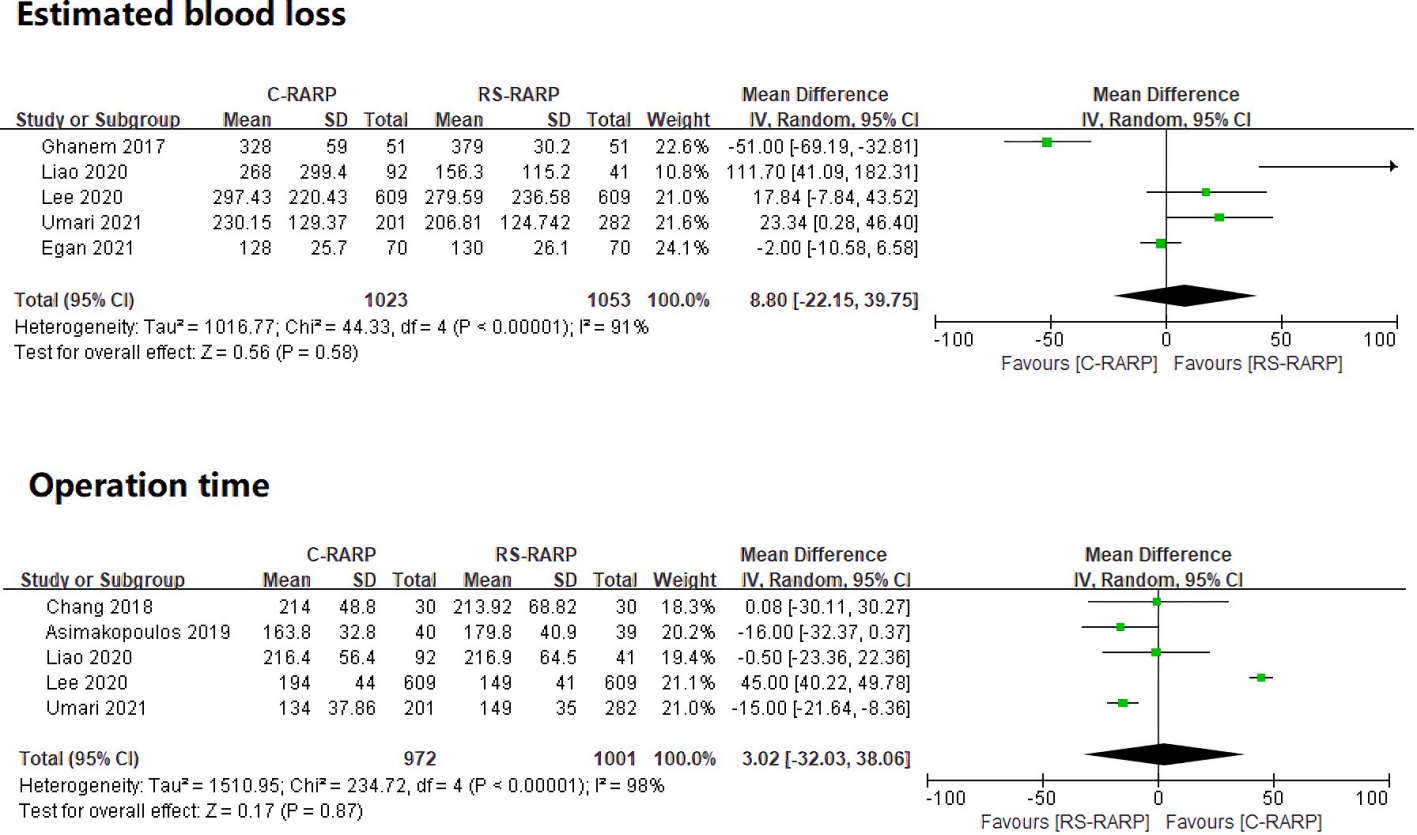
Forest plots of estimated blood loss and operation time.

EBL and operation time analyses were conducted of only studies that reported mean values and standard deviation values. A total of five studies [22, 27–30] were included in the EBL analysis. The result was WMD = 8.80; 95% CI, -22.15 to 39.75; p = 0.58; I^2^ = 91%. There were no statistically significant intergroup differences, and heterogeneity was observed. A total of five studies [24, 25, 27–29] were included in the operation time analysis. The result was WMD = 3.02; 95% CI, -32.03 to 38.06; p = 0.87; I^2^ = 98%. There was also no statistically significant intergroup difference, and heterogeneity was observed (Fig 5).

#### 3.3.4 Complications

**Fig 6:**
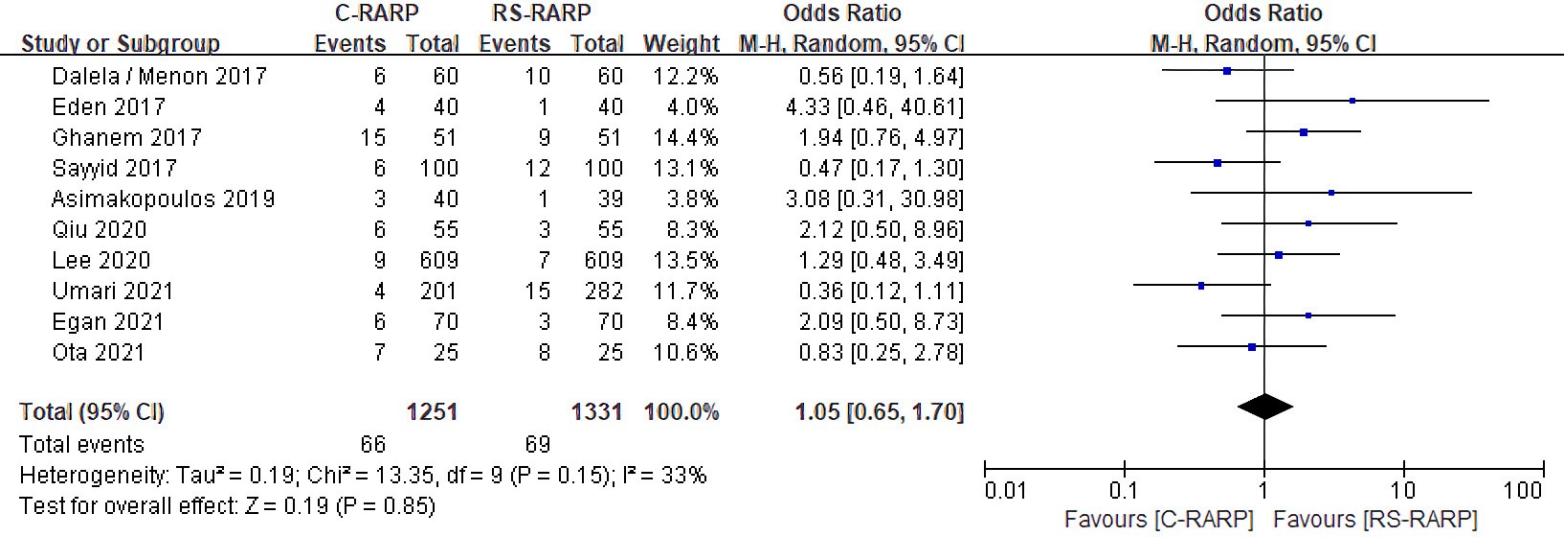
Forest plots of complications.

A total of 10 studies [19–23, 25, 26, 28–30, 32] were included in the complications analysis. Complications were classified according to Clavien–Dindo classification; the study by Lee et al. [28] reported only grade 3 or higher complications, while the remaining studies were reported as total complications. The result of the meta-analysis on the occurrence of complications was OR = 1.05; 95% CI, 0.65–1.70; p = 0.85; I^2^ = 33%. There were no statistically significant intergroup differences, and no heterogeneity was observed (Fig 6).

## 4. Discussion

RP is a standard treatment with good oncological outcomes for localized and locally advanced PCa cases. Therefore, urologists are paying close attention to reducing functional complications such as incontinence and erectile dysfunction that may occur after RP [33, 34]. The Retzius space is a structure that becomes a landmark for surgery in cases of urologic and gynecological diseases [35, 36]. The pubourethral ligaments and eventual accessory pudendal arteries within this structure may be responsible for functional outcomes [37, 38]. Therefore, RS-RARP was first introduced under the assumption that preserving the Retzius space may help prevent incontinence [9]. They presented on the superiority of continence after RS-RARP versus C-RARP [39]. Since then, several surgeons have implemented this surgical method for PCa. Although some meta-analyses have been published in recent years to provide comparative results of RS-RARP and C-RARP, debate persists [40–45]. Published studies often yield conflicting results, and the number of analyzed studies remains small. Therefore, this study included the largest number of patients to date by synthesizing recent studies. We compared and analyzed our results with meta-analyses published within the last 2 years. As it is a topic of interest to many urologists, five papers were published on the same topic from 2020 to present [40, 42–45]. Among them, Rosenberg et al. [45] performed a meta-analysis of only RCT studies, while the rest analyzed both RCTs and non-RCTs as in our study. The level of evidence could be raised by including only RCTs in the meta-analysis. However, few RCTs have been conducted on this topic. A total of five studies were included in the meta-analysis by Rosenberg et al. [45] However, two were conference data. The inclusion of conference data in meta-analyses is controversial [46–48]. Therefore, we did not include those two studies. Furthermore, given that RCT research is scarce, we combined the non-RCTs that passed the quality assessment. Our strategy is similar to other recently published meta-analyses, but most previous meta analysis studies included fewer than 10 studies [40, 43, 44]. However, we proceeded with a total of 13 studies and a larger number of patients, including studies published more recently than those included in previous meta-analyses. In particular, the study by Lim et al. [49] included in the previous meta-analyses was recently updated and published. This was analyzed for the first time in our study [28].

Most meta-analyses show a common result regarding the superiority of early continence recovery in RS-RARP. However, compared with C-RARP, late continence recovery and PSM in RS-RARP remain debatable. In RS-RARP, continence recovery is among the most important postoperative outcomes. Looking at the previous meta-analyses, all report that RS-RARP is excellent in early urinary incontinence recovery, but different results appear in long term follow-up. Study by Checcucci et al. [44] reported that 12-month long term follow up continence was also better in RS-RARP, on the other hand, studies by Barakat et al. [42], Rosenberg et al. [45] and Phukan et al. [43] reported that there was no difference between the two methods in long term continence recovery. In our study, unlike other studies, urinary incontinence was categorized and reported in two definitions. In previous studies, the definition of incontinence was not classified; however, in clinical practice, the criteria for zero pad versus safety pad are clear and useful [50]. Therefore, it is possible to analyze the two criteria separately to reflect the real clinical setting. Based on the safety pad, RS-RARP has better continence recovery than C-RARP regardless of the postoperative period. However, there is no significant difference in the recovery of late continence after 12 months based on a zero pad. The safety pad can involve a more vague definition than the zero pad [51]. Therefore, RS-RARP is better for early incontinence recovery than C-RARP; however, it is difficult to conclude that it is also better for late continence recovery using stricter criteria. Therefore, we believe that this result is more reliable than the results of previous studies on urinary incontinence recovery in RS-RARP through a more accurate analysis.

And next, in the analysis of PSM, previous meta-analyses have controversy. The Cochrane review by Rosenberg et al. [45] reported that PSM was significantly higher in RS-RARP, but the level of evidence was low. In the most recent study by Barakat et al. [42], although not a statistically significant result, it was announced that there was a significantly higher trend in RS-RARP in pT3 or higher. Also, in study by Checcucci et al. [44], when the overall patients were analyzed, RS-RARP was significantly higher, and there were no specific findings in the sub analysis. On the other hand, a study by Phukan et al. [43] reported no statistically significant difference in PSM. Our study has crucial distinction from previous studies to address these controversies. Although other studies were sub-analyzed by T stage, they did not classify the number of patients at each T stage. Therefore, many studies had inaccurate numbers because the analysis included the total patients and not the subgroups analyzed by T stage [42–44]. In the current study, sub-analyses were performed for PSM in T2 and T3 patients. As a result, there was no statistically significant differences in T2 stage or total T stage, but RS-RARP showed a higher PSM rate than C-RARP for stage T3 patients. PSM in RS-RARP was debatable because it was performed in a relatively narrow Douglas space. Our results are the first meta-analysis to statistically prove an increase in PSM in patients with T3 or higher in RS-RARP. Therefore, our results show that a more cautious approach should be taken if RS-RARP is performed in patients with locally advanced PCa. Since many studies related to RS-RARP have been conducted recently, there are few long-term follow-up reports. Therefore, in the currently available literature, the evidence level is very low for an analysis of oncologic outcomes, such as biochemical recurrence. If long-term follow-up reports are published in the near future, it will be helpful to analyze oncologic outcomes of a newly reported series. In addition, there was no difference in EBL, operation time, and complication rate, which are the perioperative factors analyzed in the current study. Similar results were also reported by other published meta-analyses.

However, this meta-analysis has some limitations. First, non-RCTs were included in the analyses. Therefore, the findings should be interpreted with caution because the evidence level might be lower than that of previous meta-analyses that included only RCTs. Second, we did not analyze erectile dysfunction as a functional outcome. There are few long-term analyses of erectile dysfunction between these two surgical methods. Therefore, previously published meta-analyses did not analyze erectile dysfunction or found no difference between the two surgical methods. In addition, the level of evidence for comparing erectile dysfunction was very low in previous studies [42, 45]. Therefore, further research on this issue should be performed. Finally, the surgeon’s experience and surgical techniques can significantly impact the study. However, in meta-analyses of differences in surgical techniques, it is difficult to reflect surgeon experience level, resulting in these limitations [52].

Despite these limitations, this study is an updated study compared to previously published studies with more accurate statistical methods and including the latest studies. Therefore, we believe that our results lead to more reliable results than previous studies. Therefore, we think that it can help readers who come across our research more accurately compare and understand RS-RARP and C-RARP.

## 5. Conclusions

Our analysis showed that RS-RARP is superior to C-RARP for early continence recovery. However, RS-RARP showed a relatively high PSM in locally advanced PCa cases of pT3 or above. Therefore, when performing RS-RARP, a cautious approach is required in high-risk local disease, although it has advantages in functional outcome. However, these results should be interpreted with caution, as there are inherent limitations of studies that include retrospective studies, until well-designed long-term follow-up multicenter RCTs are published.

## Supporting information

S1 Checklist PRISMA 2020 checklist. (DOCX)

S1 Fig Funnel plot of positive surgical margin. (PNG)

S1 Table Search strategies for systematic review. (DOCX)
