# Supplementary figures and images for: Outcomes of Retzius-sparing versus conventional robot-assisted radical prostatectomy: A KSER update series systematic review and meta-analysis
Source: PLoS One. 2022 May 26;17(5):e0268182. doi: 10.1371/journal.pone.0268182 (PMC9135208; doi:10.1371/journal.pone.0268182)

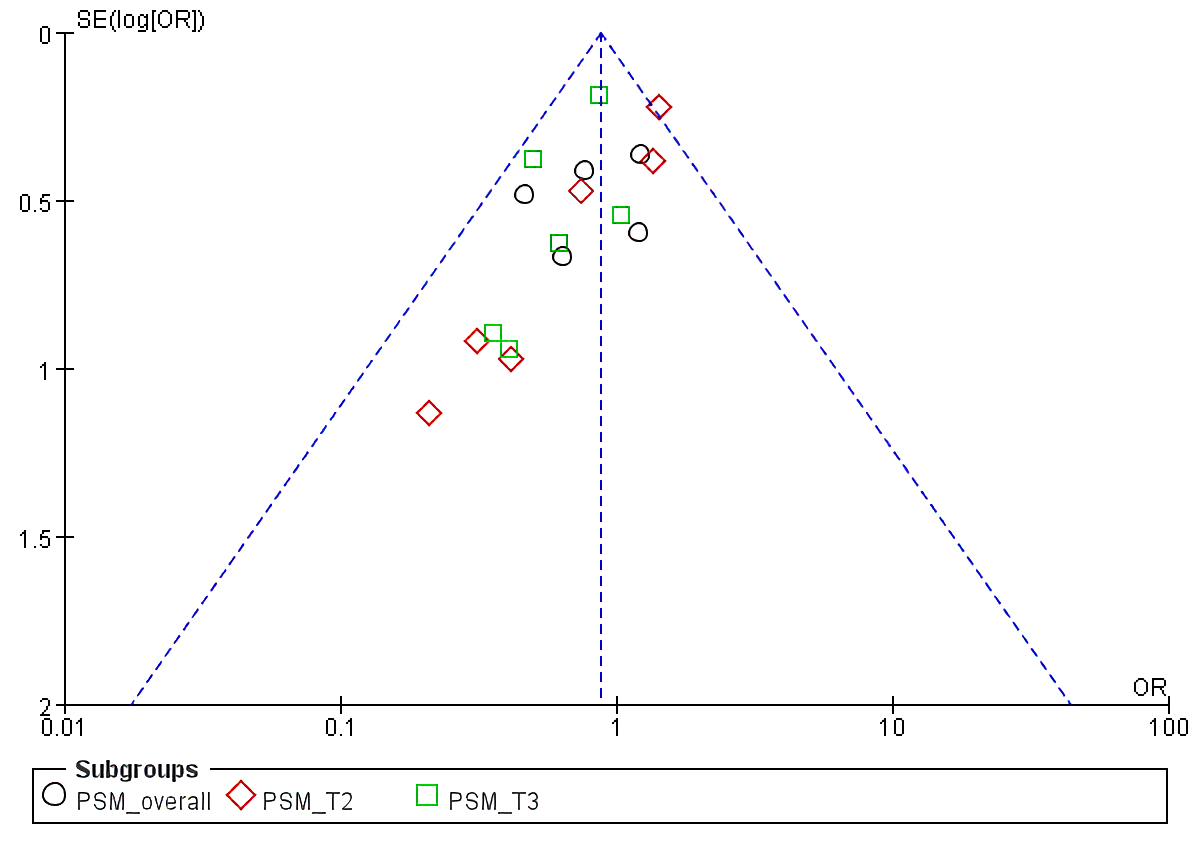

Supplement: S1 Fig — (PNG) [file pone.0268182.s002.png]
