# Supplementary material for: Outcomes of Retzius-sparing versus conventional robot-assisted radical prostatectomy: A KSER update series systematic review and meta-analysis
Source: PLoS One. 2022 May 26;17(5):e0268182. doi: 10.1371/journal.pone.0268182 (PMC9135208; doi:10.1371/journal.pone.0268182)
Supplement: S1 Table — (DOCX) [file pone.0268182.s003.docx]

1. **PubMed (**No. of article : 100)

"retzius"[All Fields] AND ("spare"[All Fields] OR "spared"[All Fields] OR "spares"[All Fields] OR "sparing"[All Fields]) AND ("prostatectomy"[MeSH Terms] OR "prostatectomy"[All Fields] OR "prostatectomies"[All Fields])

1. **OVID-EMBASE**

| **OVID-EMBASE** | | |
| --- | --- | --- |
| No. | Search term | No. of article |
| #1 | retzius.m_titl. | 600 |
| #2 | sparing.m_titl. | 15144 |
| #3 | #1 and #2 | 147 |
| #4 | prostatectomy.m_titl. | 25944 |
| #5 | #3 and #4 | 138 |

1. **Cochrane library**

| **Cochrane library** | | |
| --- | --- | --- |
| No. | Search term | No. of article |
| #1 | retzius* | 56 |
| #2 | sparing* | 5904 |
| #3 | #1 and #2 | 34 |
| #4 | prostatectomy* | 4637 |
| #5 | #3 and #4 | 33 |
